# Adverse Health Outcomes Among Industrial and Occupational Sectors in Michigan

**DOI:** 10.5888/pcd15.170487

**Published:** 2018-08-09

**Authors:** Ling Wang, Kenneth Rosenman

**Affiliations:** 1 Department of Medicine, Michigan State University, East Lansing, Michigan

## Abstract

**Introduction:**

We used data from the Michigan Behavioral Risk Factor Surveillance System (MIBRFSS) to estimate the prevalence of adverse health outcomes by industry and occupation and to examine the association of adverse health outcomes with industry and occupation while controlling for demographics and personal lifestyle behaviors.

**Methods:**

We calculated the prevalence of adverse health outcomes by industry by using data from the 2013–2015 MIBRFSS. Adjusted prevalence of adverse health outcomes was calculated by industry and occupation by using logistic regression for survey design, adjusting for demographics and health behaviors, and was compared with the overall prevalence in all industries and occupations.

**Results:**

Three industries had a significantly higher prevalence of current asthma, diabetes, and depression compared with prevalence among workers employed in all industries. After controlling for confounding factors, only Health Care and Social Assistance had significantly higher prevalence of a health outcome, depression (20.1%). Three occupations had significantly higher prevalence of chronic obstructive pulmonary disease, current asthma, depression, high blood pressure, and diabetes compared with workers employed in all occupations. After adjusting for all confounding factors, only one occupation, protective service, had a significantly higher prevalence of high blood pressure (37.3%) and diabetes (12.8%).

**Conclusion:**

Adverse health outcomes varied significantly by industry and occupation in Michigan. Employers, policy makers, and health promotion practitioners can use results based on BRFSS to target and prioritize worksite wellness programs. MIBRFSS data also suggested the need for further research to identify why some industries had higher risks for diabetes, hypertension, and depression after controlling for covariates.

## Introduction

The national Behavioral Risk Factor Surveillance System (BRFSS) is an annual telephone survey of adults aged 18 or older that is conducted independently by the states, the District of Columbia, and US territories and is coordinated through cooperative agreements with the Centers for Disease Control and Prevention (CDC). The survey comprises 3 parts: core survey questions, optional modules, and state-added questions. These state-based surveys provide state-specific, population-based estimates of the prevalence of various behaviors, medical conditions, and preventive health care practices by state (1). For many of these topics, BRFSS is the main source of state-level prevalence information, and BRFSS data are used to set and track national health objectives such as *Healthy People 2020* (2). The Michigan Behavioral Risk Factor Surveillance System (MIBRFSS) follows the CDC telephone survey protocol for BRFSS and uses the annual standardized core questionnaire. In addition, MIBRFSS includes about 25 state-added questions each year.

We examined the prevalence of health risk factors and chronic health conditions by industry and occupation in Michigan by using 2013–2015 MIBRFSS data. Previous publications in other states have compared the prevalence of health status or risk factors by industry and occupation, but many have not controlled for confounding factors such as age, sex, race, and smoking behaviors (3–5), although some publications have controlled for covariates (6–8). In our analysis, we examined unadjusted prevalence and then used logistic regression for complex surveys to predict the adjusted prevalence of adverse health outcome by industry and occupation, controlling for other risk factors. Ours is the first study in Michigan to use BRFSS data to estimate the prevalence of adverse health outcomes by industry and occupation, and it provides insights on work-related health differences in Michigan.

## Methods

In 2013, 2014, and 2015, MIBRFSS included the 2 standardized questions for a respondent’s current or most recent job that were developed by the National Institute for Occupational Safety and Health (NIOSH) in cooperation with the states: 1) Industry – “What kind of business or industry do you work in, for example, hospital, elementary school, clothing manufacturing, restaurant?”; 2) Occupation – “What kind of work do you do, for example, registered nurse, janitor, cashier, auto mechanic?” All respondents who were employed for wages, self-employed, or out of work for less than one year at the time of their interview were defined as employed and were asked the 2 occupation and industry questions. Participants’ responses were coded to 2002 US Census Bureau industry and occupation numeric codes using the NIOSH Industry and Occupation Computerized Coding System and human coders. Census industry codes were grouped into the 20 Standard Industrial Classification (SIC) 2002 codes and 22 Standard Occupational Classification (SOC) 2000 codes for major groups. The industrial and the occupational category for military personnel were excluded from analyses because BRFSS data are not representative of those personnel.

Data were weighted on the basis of an iterative proportional fitting methodology (also known as “raking” weighting) to adjust for the distribution of the Michigan adult population by telephone source (landline or cellular phone), race/ethnicity, education level, marital status, age by sex, sex by race/ethnicity, age by race/ethnicity, and renter/homeowner status. To determine the prevalence of health outcomes by industry or occupation, we obtained weighted prevalence and 95% confidence intervals (CIs) based on the BRFSS sample survey design. Some industries’ results were suppressed following the BRFSS data guidelines: an unweighted denominator of fewer than 50 respondents or a relative standard error of greater than 30%. Results from 2 of the 20 industries, Management and Mining, were suppressed. For occupation, results were presented from 13 grouped occupations. Logistic regressions based on complex survey design were employed to predict the prevalence of different adverse health outcomes in different industries and occupations (7–9). All predicted prevalences in the regression analysis were adjusted for respondents’ age, sex, race, body mass index (height in m^2^ /weight in kilograms), smoking status, education level, income, exercise, binge drinking, and health insurance status. For each health outcome, we also obtained predicted prevalence for workers in all industry and occupation groups and compared this with prevalence in each industry and occupation.

We performed analyses by using SAS (SAS Institute, Inc)–callable SUDAAN (RTI International) and STATA (STATA Corp) software, and analyses were weighted to adjust for MIBRFSS survey sampling design. We considered the adjusted prevalence estimates to differ statistically if their 95% CIs did not overlap. This is approximately equivalent to setting the type I error for the null hypothesis at α = 0.006 level (10).

The 2 outcomes addressed were 1) were there significant differences in the prevalence of adverse health outcomes in different industry and occupation groups compared with all employed workers and 2) were there significant differences in the prevalence of adverse health outcomes in different industry or occupation groups after controlling for confounding factors. MIBFRSS is de-identified and therefore exempt from institutional review board approval.

## Results

### Respondent demographics

Ninety-four percent of respondents completed the occupation questions, and 97% completed the industry questions. Manufacturing (18.8%) and Health Care and Social Assistance (15.7%) were the 2 most common industries in which respondents were employed (worked in the previous year). We assessed the distribution of demographic characteristics among all employed adults by industry (Table 1). Because of hiring practices, societal norms, differences in educational attainment, and work requirements of each industry, the demographic distribution differed markedly among industries. For example, an average of 53.3% of the employed population were men; in Construction, 90.3% were men, and in Health Care And Social Assistance, 19.3% were men.

**Table 1:** Distribution of Demographic Characteristics Among Employed Adults by Industry, Michigan Behavioral Risk Factor Surveillance System (MIBRFSS), 2013–2015

Standard Industrial Classification, Code	Race	% (95% CI)	Age, y	% (95% CI)	Education	% (95% CI)	Sex	% (95% CI)
**Accommodation and Food Services, 72**	White	72.5 (68.1–76.8)	18–24	40.4 (35.8–45.0)	<HS	14.1 (9.9–18.4)	Male	47.1 (42.6–51.6)
	Black	16.1 (12.6–19.7)	25–34	23.7 (19.5–27.8)	HS graduate	39 (34.6–43.4)	NA	NA
	Other	5.0 (3.0–6.9)	35–44	15.0 (11.6–18.3)	Some college	39.5 (35.0–44.0)	NA	NA
	Hispanic	6.5 (3.6–9.3)	45–54	12.4 (9.7–15.1)	College graduate	7.4 (5.6–9.2)	NA	NA
	Not applicable	NA	55–64	6.8 (5.0–8.5)	NA	NA	NA	NA
	Not applicable	NA	65–74	1.4 (0.7–2.0)	NA	NA	NA	NA
**Administrative, Support, and Waste Management, 56**	White	72.1 (65.9–78.3)	18–24	20.9 (14.9–26.8)	<HS	15.0 (9.4–20.5)	Male	53.0 (46.6–59.3)
	Black	15.8 (11.0–20.6)	25–34	23.4 (17.8–29.0)	HS graduate	37.9 (31.6–44.2)	NA	NA
	Other	3.9 (1.8–6.0)	35–44	20.6 (15.0–26.2)	Some college	33.0 (26.6–39.3)	NA	NA
	Hispanic	8.2 (3.6–12.7)	45–54	20.4 (15.7–25.1)	College graduate	14.2 (10.8–17.6)	NA	NA
	Not applicable	NA	55–64	11.9 (8.2–15.7)	NA	NA	NA	NA
	Not applicable	NA	65–74	2.3 (1.1–3.6)	NA	NA	NA	NA
**Agriculture, Forestry, Fishing, and Hunting, 11**	White	95.4 (91.5–99.3)	18–24	19.4 (12.0–26.8)	<HS	16.1 (8.7–23.5)	Male	83.4 (77.8–89.1)
	Other	2.3 (0–5.2)	25–34	19.1 (12.0–26.2)	HS graduate	44.8 (36.3–53.3)	NA	NA
	Hispanic	2.3 (0–5.0)	35–44	16.9 (10.3–23.4)	Some college	25.7 (18.8–32.6)	NA	NA
	Not applicable	NA	45–54	16.8 (10.1–23.4)	College graduate	13.4 (8.9–17.8)	NA	NA
	Not applicable	NA	55–64	17.0 (11.8–22.2)	NA	NA	NA	NA
	Not applicable	NA	65–74	7.2 (4.1–10.3)	NA	NA	NA	NA
**Arts, Entertainment, and Recreation, 71**	White	79.7 (72.6–86.8)	18–24	21.4 (14.2–28.7)	<HS	5.0 (0.1–9.9)	Male	64.0 (56.8–71.2)
	Black	11.3 (5.4–17.2)	25–34	16.7 (10.3–23.0)	HS graduate	33.9 (26.2–41.6)	NA	NA
	Other	6.6 (2.6–10.6)	35–44	20.1 (13.4–26.9)	Some college	36.9 (29.7–44.2)	NA	NA
	Hispanic	2.4 (0–5.3)	45–54	16.1 (10.8–21.5)	College graduate	24.2 (18.6–29.7)	NA	NA
	Not applicable	NA	55–64	16.8 (11.9–21.7)	NA	NA	NA	NA
	Not applicable	NA	65–74	6.0 (3.4–8.6)	NA	NA	NA	NA
**Construction, 23**	White	86.7 (83.6–89.8)	18–24	10.6 (7.6–13.6)	<HS	16.1 (11.8–20.5)	Male	90.3 (87.7–92.8)
	Black	5.6 (3.5–7.7)^a^	25–34	23.8 (19.5–28.1)	HS graduate	38.6 (34.4–42.7)	NA	NA
	Other	2.9 (1.4–4.4)	35–44	25.1 (21.1–29.1)	Some college	33.0 (28.9–37.1)	NA	NA
	Hispanic	4.8 (2.7–6.8)	45–54	23.0 (19.5–26.5)	College graduate	12.3 (10.1–14.5)	NA	NA
	Not applicable	NA	55–64	14.1 (11.7–16.5)	NA	NA	NA	NA
	Not applicable	NA	65–74	3.1 (2.1–4.0)	NA	NA	NA	NA
**Educational Services, 61**	White	81.5 (78.8–84.3)	18–24	7.0 (5.1–9.0)	<HS	1.5 (0.2–2.8)	Male	30.2 (27.3–33.1)
	Black	10.8 (8.5–13.0)	25–34	14.6 (12.3–17.0)	HS graduate	15.1 (12.7–17.5)	NA	NA
	Other	3.9 (2.7–5.1)	35–44	25.6 (22.9–28.4)	Some college	23.5 (20.7–26.3)	NA	NA
	Hispanic	3.8 (2.3–5.4)	45–54	26.2 (23.6–28.8)	College graduate	59.8 (56.7–63.0)	NA	NA
	Not applicable	NA	55–64	22.3 (20.0–24.5)	NA	NA	NA	NA
	Not applicable	NA	65–74	3.7 (2.9–4.5)	NA	NA	NA	NA
**Finance and Insurance, 52**	White	83.4 (79.6–87.1)	18–24	8.3 (5.2–11.4)	<HS	0.5 (0–1.5)	Male	38.3 (33.5–43.2)
	Black	10.0 (7.0–13.0)	25–34	24.0 (19.1–28.9)	HS graduate	19.4 (15.0–23.7)	NA	NA
	Other	3.4 (1.8–5.0)	35–44	19.5 (15.6–23.5)	Some college	36.6	NA	(31.8–41.5)
	Hispanic	3.2 (1.2–5.2)	45–54	26.5 (22.3–30.6)	College graduate	43.5 (38.8–48.2)	NA	NA
	Not applicable	NA	55–64	15.4 (12.5–18.2)	NA	3.6 (2.1–5.0)	Male	NA
	Not applicable	NA	65–74	5.5 (3.6–7.5)	NA	NA	NA	NA
**Health Care and Social Assistance, 62**	White	75.8 (73.6–78.1)	18–24	8.7 (7.2–10.2)	<HS	NA	NA	19.3 (17.3–21.2)
	Black	14.8 (12.8–16.8)	25–34	22.3 (20.1–24.6)	HS graduate	17.1 (15.2–19.0)	NA	NA
	Other	5.5 (4.3–6.6)	35–44	22.8 (20.6–25)	Some college	39.8 (37.4–42.3)	NA	NA
	Hispanic	3.9 (2.8–5.1)	45–54	23.4 (21.5–25.3)	College graduate	39.5 (37.3–41.7)	NA	NA
	Not applicable	NA	55–64	18.6 (17–20.2)	NA	NA	NA	NA
	Not applicable	NA	65–74	3.4 (2.8–4.0)	NA	3.8 (0.6–7.0)	Male	NA
**Information, 51**	White	86.5 (81.7–91.3)	18–24	12.1 (6.1–18.0)	<HS	NA	NA	65.6 (59.0–72.2)
	Black	8.6 (4.7–12.5)	25–34	24.4 (17.6–31.2)	HS graduate	20.1 (14.0–26.2)	NA	NA
	Other	3.5 (0.9–6.1)	35–44	19.0 (13.5–24.4)	Some college	40.9 (33.5–48.2)	NA	NA
	Hispanic	1.4 (0–3.2)	45–54	27.0 (20.8–33.1)	College graduate	35.2 (29.0–41.4)	NA	NA
	Not applicable	NA	55–64	14.1 (9.7–18.4)	NA	NA	NA	NA
	Not applicable	NA	65–74	2.6 (0.9–4.3)	NA	NA	NA	NA
**Manufacturing, 31**	White	78.4 (76.0–80.7)	18–24	10.0 (8.3–11.6)	<HS	9.1 (7.1–11.2)	Male	73.6 (71.5–75.7)
	Black	12.4 (10.4–14.4)	25–34	20.1 (17.9–22.3)	HS graduate	33.5 (31.2–35.9)	NA	NA
	Other	4.7 (3.6–5.8)	35–44	22.3 (20.1–24.5)	Some college	33.1 (30.8–35.5)	NA	NA
	Hispanic	0.5 (3.3–5.8)	45–54	27.6 (25.5–29.7)	College graduate	24.2 (22.4–26.0)	NA	NA
	Not applicable	NA	55–64	17.7 (16.1–19.3)	NA	NA	NA	NA
	Not applicable	NA	65–74	2.0 (1.5–2.5)	NA	NA	NA	NA
**Other Services (Except Public Administration), 81**	White	73.6 (68.9–78.3)	18–24	12.3 (8.2–16.4)	<HS	10.4 (6.1–14.7)	Male	51.5 (46.6–56.3)
	Black	16.5 (12.5–20.6)	25–34	22.3 (17.7–26.8)	HS graduate	30.2 (25.8–34.7)	NA	NA
	Other	6.3 (3.6–9.1)	35–44	19.2 (15.4–22.9)	Some college	35.2 (30.6–39.8)	NA	NA
	Hispanic	3.5 (1.5–5.6)	45–54	24.3 (20.5–28.0)	College graduate	24.1 (20.6–27.6)	NA	NA
	Not applicable	NA	55–64	15.6 (12.9–18.3)	NA	NA	NA	NA
	Not applicable	NA	65–74	5.3 (3.8–6.7)	NA	NA	NA	NA
**Professional, Scientific, and Technical Service, 54**	White	82.4 (78.8–86.1)	18–24	5.8 (3.5–8.0)	<HS	1.7 (0–3.5)	Male	56.3 (52.0–60.7)
	Black	7.9 (5.3–10.5)	25–34	26.8 (22.7–31.0)	HS graduate	10.3 (7.2–13.4)	NA	NA
	Other	6.1 (3.9–8.3)	35–44	21.8 (17.8–25.7)	Some college	33.7 (29.1–38.2)	NA	NA
	Hispanic	3.6 (1.5–5.8)	45–54	23.4 (19.8–26.9)	College graduate	54.3 (49.8–58.9)	NA	NA
	Not applicable	NA	55–64	16.7 (14–19.4)	NA	NA	NA	NA
	Not applicable	NA	65–74	4.7 (3.5–5.9)	NA	NA	NA	NA
**Public Administration, 56**	White	76.0 (71.8–80.2)	18–24	5.0 (2.0–8.1)	<HS	2.3 (0–4.9)	Male	51.9 (47.1–56.7)
	Black	15.9 (12.2–19.6)	25–34	19.4 (15.1–23.7)	HS graduate	13.7 (10.5–16.8)	NA	NA
	Other	3.2 (1.7–4.8)	35–44	25.4 (21.1–29.8)	Some college	41.7 (36.8–46.6)	NA	NA
	Hispanic	4.9 (2.6–7.2)	45–54	26.3 (22.3–30.3)	College graduate	42.3 (37.7–46.9)	NA	NA
	Not applicable	NA	55–64	20.0 (16.8–23.3)	NA	NA	NA	NA
	Not applicable	NA	65–74	3.0 (2.0–4.1)	NA	NA	NA	NA
**Real Estate, Rental, and Leasing, 53**	White	81.2 (74.5–88.0)	18–24	10.6 (5.5–15.7)	<HS	6.9 (1.4–12.5)	Male	50.8 (43.5–58.1)
	Black	12.1 (6.0–18.1)	25–34	19.5 (12.4–26.7)	HS graduate	21.2 (15.4–26.9)	NA	NA
	Other	4.7 (1.7–7.7)	35–44	14.7 (9.0–20.4)	Some college	44.0 (36.4–51.7)	NA	NA
	Hispanic	2.0 (0–4.6)	45–54	22.0 (15.8–28.2)	College graduate	27.9 (21.7–34.0)	NA	NA
	Not applicable	Not applicable	55–64	23.0 (17.4–28.6)	NA	NA	NA	NA
	Not applicable	Not applicable	65–74	7.6 (4.8–10.4)	NA	NA	NA	NA
**Retail Trade, 44**	White	84.7 (82.0–87.3)	18–24	21.7 (18.4–24.9)	<HS	7.3 (4.5–10.1)	Male	49.1 (45.6–52.6)
	Black	8.7 (6.6–10.8)^a^	25–34	20.9 (17.8–24.0)	HS graduate	37.4 (34.1–40.7)	NA	NA
	Other	2.9 (1.9–4.0)	35–44	16.6 (14.0–19.2)	Some College	NA	NA	NA
	Hispanic	3.7 (2.1–5.4)	45–54	20.0 (17.6–22.5)	College graduate	17.7 (15.6–19.7)	NA	NA
	Not applicable	Not applicable	55–64	15.8 (13.8–17.8)	NA	NA	NA	NA
	Not applicable	Not applicable	65–74	3.7 (2.9–4.5)	NA	NA	NA	NA
**Transportation and Warehousing, 48**	White	77.2 (72.6–81.7)	18–24	8.6 (5.0–12.3)	<HS	5.7 (2.1–9.3)	Male	76.5 (72.0–80.9)
	Black	15.7 (11.5–19.8)	25–34	15.7 (11.3–20.2)	HS graduate	38.9 (33.6–44.2)	NA	NA
	Other	4.2 (2.1–6.4)	35–44	21.7 (17.0–26.4)	Some college	41.3 (35.8–46.8)	NA	NA
	Hispanic	2.9 (0.9–4.9)	45–54	29.9 (24.9–34.9)	College graduate	14.2 (11.0–17.3)	NA	NA
	Not applicable	NA	55–64	21.1 (17.3–24.9)	NA	NA	NA	NA
	Not applicable	NA	65–74	2.4 (1.4–3.5)	NA	NA	NA	NA
**Utilities, 22**	White	78.4 (70.7–86.1)	18–24	6.9 (1.2–12.6)	<HS	3.3 (0–8.4)	Male	85.4 (79.5–91.3)
	Black	11.8 (5.1–18.6)	25–34	15.6 (7.9–23.3)	HS graduate	21.5 (13.6–29.3)	NA	NA
	Other	6.5 (2.6–10.3)	35–44	18.5 (10.6–26.4)	Some college	51.3 (41.6–60.9)	NA	NA
	Hispanic	3.3 (1.1–5.4)	45–54	34.1 (25.5–42.7)	College graduate	24.0 (17.8–30.2)	NA	NA
	Not applicable	NA	55–64	21.2 (13.8–28.6)	NA	NA	NA	NA
	Not applicable	NA	65–74	2.5 (0.2–4.8)	NA	NA	NA	NA
**Wholesale and Trade, 42**	White	81.8 (74.9–88.7)	18–24	7.9 (4.1–11.8)	<HS	0.8 (0–2.0)	Male	68.0 (61.3–74.7)
	Black	5.9 (1.0–10.9)^a^	25–34	15.1 (8.5–21.8)	HS graduate	33.2 (26.1–40.4)	NA	NA
	Other	6.3 (2.6–10.1)	35–44	20.8 (14.6–27.1)	Some college	38.5 (30.9–46.1)	NA	NA
	Hispanic	5.9 (1.6–10.3)	45–54	28.8 (22.3–35.3)	College graduate	27.4 (21.4–33.4)	NA	NA
	Not applicable	NA	55–64	20.8 (15.7–25.9)	NA	NA	NA	NA
	Not applicable	NA	65–74	5.6 (3.4–7.7)	NA	NA	NA	NA
**All**	White	79.3 (78.4–80.3)	18–24	13.0 (12.2–13.8)	<HS	7.2 (6.3–8.0)	Male	53.3 (52.2–54.3)
	Black	11.9 (11.2–12.7)	25–34	20.9 (20.0–21.9)	HS graduate	27.9 (26.9–28.8)	NA	NA
	Other	4.5 (4.1–4.9)	35–44	21.1 (20.3–22.1)	Some college	35.7 (34.7–36.7)	NA	NA
	Hispanic	4.2 (3.7–4.7)	45–54	23.8 (22.9–24.6)	College graduate	29.2 (28.4–30.1)	NA	NA
	Not applicable	NA	55–64	16.9 (16.3–17.6)	NA	NA	NA	NA
	Not applicable	NA	65–74	3.4 (3.1–3.6)	NA	NA	NA	NA

Abbreviations: HS, high school; NA, not available.

a Significant difference compared with employed workers in all industries in the survey.

Arts, Entertainment, and Recreation had a significantly younger population (21.4% were aged 18 to 24). In Public Administration, most of the employed population were aged 35 to 64 (over 70%), which was significantly higher than the overall proportion of the employed population in this age range (Table 1).

For racial/ethnic distribution, we found no notable differences in the Hispanic population by industry. A significantly low proportion of African Americans worked in Construction and in Wholesale and Retail Trade, and no African Americans reported working in Agriculture, Forestry, Fishing, and Hunting (Table 1).

### Risk factors

We examined the prevalence of binge drinking, current cigarette smoking, and obesity by industry (Figure). The average prevalence of current cigarette smoking among employed adults was 22.1% with the highest prevalence in construction (36.3%) and the lowest prevalence in educational services (8.8%). Three industries, including Construction, had a significantly elevated prevalence of current smoking, and 7 industries, including education, had a significantly lower prevalence of current smoking compared with the average smoking rate of workers in all industries.

**Figure:**
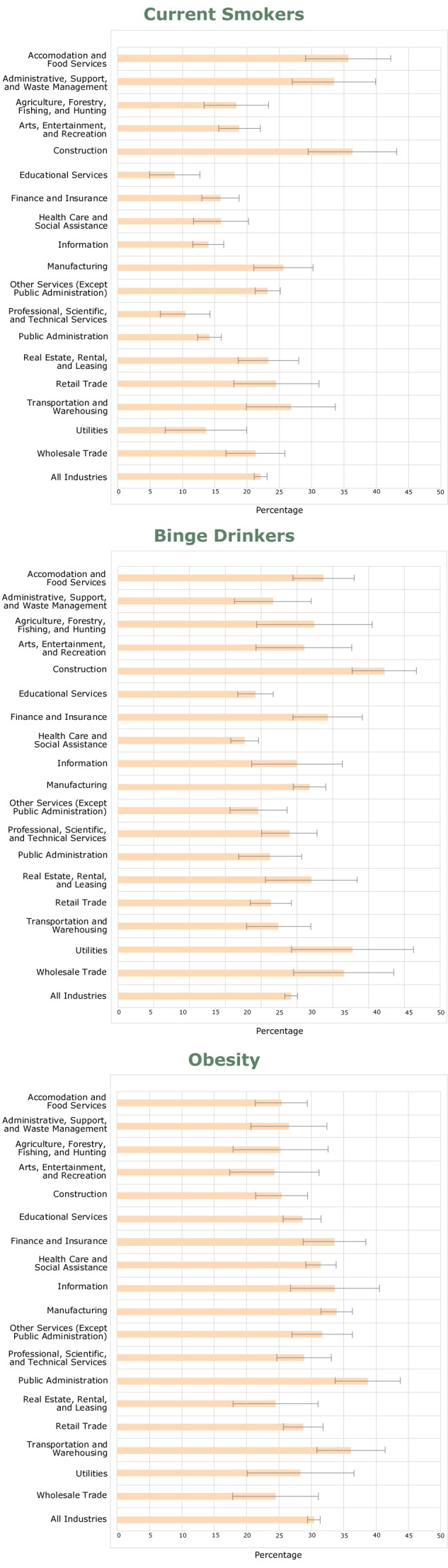
Prevalence of current smokers, binge drinkers, and obesity among employed adults, by industry, Michigan Behavioral Risk Factor Surveillance System, 2013–2015. Brackets represent the 95% confidence intervals (CIs), and nonoverlapping 95% CIs indicate a significantly different prevalence compared with all workers in the survey (*P* < .05). **Table:** 

Industry or Occupation	Current Smoker, % (95% CI)	Binge Drinker, % (95% CI)	Obesity, % (95% CI)
Accommodation and Food Services	35.7 (31.1–40.2)	28.7 (24.4–33.0)	25.4 (21.4–29.4)
Administrative and Support and Waste Management	33.5 (27.2–39.8)	21.7 (16.3–27.0)	26.5 (20.6–32.4)
Agriculture, Forestry, Fishing and Hunting	18.3 (11.4–25.2)	27.4 (19.4–35.5)	25.2 (17.9–32.6)
Arts, Entertainment, and Recreation	18.8 (12.3–25.4)	26.0 (19.3–32.7)	24.3 (17.4–31.2)
Construction	36.3 (31.6–41)	37.2 (32.7–41.7)	25.4 (21.4–29.4)
Educational Services	8.8 (7–10.7)	19.2 (16.7–21.7)	28.6 (25.7–31.5)
Finance and Insurance	15.9 (12–19.8)	29.3 (24.4–34.2)	33.6 (28.7–38.4)
Health Care and Social Assistance	16.0 (14.1–17.9)	17.7 (15.8–19.6)	31.5 (29.1–33.8)
Information	14.0 (9.4–18.6)	25.0 (18.7–31.3)	33.7 (26.8–40.5)
Manufacturing	25.6 (23.3–28)	26.8 (24.5–29.0)	33.9 (31.5–36.4)
Other Services (except Public Administration)	23.2 (19–27.4)	19.6 (15.7–23.6)	31.7 (27.0–36.3)
Professional, Scientific, and Technical Services	10.4 (7.6–13.3)	23.9 (20.1–27.8)	28.9 (24.7–33.1)
Public Administration	14.2 (10.3–18.1)	21.3 (16.9–25.6)	38.7 (33.7–43.8)
Real Estate and Rental and Leasing	23.3 (16.5–30.1)	27.0 (20.6–33.4)	24.5 (17.9–31.1)
Retail Trade	24.5 (21.3–27.8)	21.4 (18.5–24.3)	28.8 (25.7–31.8)
Transportation and Warehousing	26.8 (21.8–31.8)	22.5 (17.9–33.0)	36.1 (30.9–41.4)
Utilities	13.6 (7.2–20.1)	32.8 (24.4–27.0)	28.3 (20.1–36.6)
Wholesale Trade	21.3 (14.7–27.9)	31.6 (16.3–35.5)	24.5 (17.8–31.1)
All workers	22.1 (31.1–40.2)	24.2 (19.4–32.7)	30.4 (21.4–29.4)

Workers in Construction had the highest prevalence (37.2%) of binge drinking, which was significantly higher than the average prevalence of binge drinking among all employed adults (24.2%). Compared with the average binge-drinking prevalence among all workers, 3 industries — Educational Services, Health Care and Social Assistance and Other Services had a significantly lower prevalence of binge drinking; Health Care and Social Assistance had the lowest prevalence (17.7%) (Figure). Public Administration (38.7%) and Manufacturing (33.9%) had significantly more obese workers compared with all workers (30.4%), whereas the prevalence in Construction (25.4%) and Accommodation and Food Services (25.4%) was significantly lower.

### Health outcomes

We assessed by industry the prevalence of ever having asthma, cancer, chronic obstructive pulmonary disease (COPD), depression, diabetes, high blood pressure, and current asthma. Seven industries had a health outcome prevalence that was significantly greater for ever having asthma, cancer, COPD, depression, diabetes, and high blood pressure and for having current asthma than all industries combined, and 12 had a prevalence that was significantly lower (Table 2). After controlling for demographic information and risk behaviors only the prevalence of depression for Health Care and Social Assistance remained significantly higher than all industries combined (Table 3). Controlling for the increased prevalence of cigarette smoking among construction workers, the prevalence of ever having COPD in Construction was significantly lower than among all workers.

**Table 2:** Distribution of Health Outcomes Among Employed Adults by Industry, Michigan Behavioral Risk Factor Surveillance System (MIBRFSS), 2013–2015

Standard Industrial Classification, Code	COPD Ever	Current Asthma	Asthma Ever	Depression Ever	High Blood Pressure Ever^a^	Diabetes Ever	Any Cancer Ever
	% (95% Confidence Interval)						
**Accommodation and Food Services, 72**	6.8 (4.5–9.1)	12.8 (9.7–15.9)^b^	20.9 (17.0–24.7)^b^	24.0 (20.0–28.0)^b^	15.4 (11.9–18.9)^b^	3.6 (2.2–4.9)^b^	3.0 (1.7–4.4)^b^
**Administrative, Support, and Waste Management, 56**	4.4 (2.7–6.2)	8.2 (4.9–11.4)	12.8 (8.8–16.8)	19.1 (14.4–23.8)	21.6 (15.3–28.0)	5.7 (2.5–9.0)	5.6 (3.2–7.9)
**Agriculture, Forestry, Fishing and Hunting, 11**	NA	NA	NA	6.5 (2.4–10.5)^b^	23.6 (16.0–31.1)	1.8 (0.9–2.8)^b^	5.5 (2.4–8.7)
**Arts, Entertainment, and Recreation, 71**	NA	10.7 (6.5–15)	14.2 (9.3–19.1)	16.9 (10.7–23.0)	16.9 (10.7–23.1)^b^	12.0 (7.2–16.8)^b^	11.1 (6.8–15.5)
**Construction, 23**	2.6 (1.6–3.6)^b^	5.4 (3.3–7.6)^b^	10.5 (7.6–13.4)	11.0 (8.3–13.6)^b^	23.8 (19.4–28.2)	4.1 (2.7–5.4)	4.9 (3.3–6.5)
**Educational Services, 61**	3.4 (2.2–4.6)	12.2 (10.2–14.2)	16.3 (14.0–18.5)	15.0 (13.0–17.1)	26.9 (23.8–30.0)	5.3 (4.1–6.5)	8.6 (7.1–10.1)
**Finance and Insurance, 52**	4.1 (2.1–6)	9.7 (6.5–12.9)	14.9 (11.3–18.6)	17.8 (13.8–21.9)	28.3 (23.1–33.4)	6.9 (4.7–9.1)	9.2 (6.6–11.8)
**Health Care and Social Assistance, 62**	4.3 (3.3–5.3)	11.1 (9.5–12.6)^b^	15.4 (13.6–17.1)	22.0 (19.9–24.0)^b^	24.6 (22.3–27.0)	6.1 (5.0–7.2)	8.6 (7.3–9.8)^b^
**Information, 51**	NA	10.6 (6.6–14.5)	17.8 (12.4–23.3)	19.5 (13.6–25.5)	24.5 (17.9–31.1)	4.3 (2.1–6.6)	6.1 (2.9–9.3)
**Manufacturing, 31**	4.6 (3.5–5.6)	6.4 (5.2–7.7)^b^	12.2 (10.5–13.9)	22.0 (12.3–31.7)	27.2 (24.7–29.8)	6.2 (5.1–7.3)	5.4 (4.5–6.3)
**Other Services (Except Public Administration), 81**	5.8 (3.9–7.7)	8.7 (5.6–11.7)	13.0 (9.6–16.3)	16.6 (13.3–19.9)	22.6 (18.4–26.7)	6 (4.1–7.8)	8.4 (6.3–10.5)
**Professional, Scientific, and Technical Service, 54**	3.5 (2.0–5.0)	9.8 (7.0–12.7)	14.5 (11.3–17.8)	12.8 (9.8–15.9)b	26.2 (21.7–30.6)	5.5 (3.6–7.5)	6.9 (5.0­–8.8)
**Public Administration, 56**	3.7 (2.1–5.2)	8.2 (5.8–10.6)	14.2 (10.5–18.0)	14.9 (11.1–18.6)	26.9 (22.3–31.4)	7.6 (5.1–10.0)	7.4 (5.4–9.4)
**Real Estate, Rental, and Leasing, 53**	NA	7.5 (3.8–11.2)	11.4 (7.3–15.6)	15.3 (10.1–20.6)	28.0 (19.9–36.1)	10.3 (5.6–15)	7.0 (3.6–10.3)
**Retail Trade, 44**	4.8 (3.3–6.2)	8.7 (6.8–10.5)	15 (12.6–17.4)	18.9 (16.2–21.7)	23.3 (20.1–26.5)	4.1 (3-5.2)^b^	7.4 (5.8–8.9)
**Transportation and Warehousing, 48**	4.7 (2.6–6.7)	6.8 (4.0–9.7)	12.6 (9.0–16.3)	9.2 (6.4–12)^b^	26.7 (21.1–32.3)	8.8 (5.6–12.0)	4.8 (2.8–6.8)
**Utilities, 22**	NA	NA	8.2 (4.1–12.4)^b^	12.6 (5.8–19.5)	31.8 (22.3–41.3)	NA	6.2 (3.4–9.0)
**Wholesale Trade, 42**	NA	8.1 (4.2–12.1)	11.5 (7.2–15.9)	12.0 (7.4–16.6)	23.9 (15.3–32.5)	6.0 (3.4–8.6)	7.9 (5-10.8)
**All Industries**	4.3 (3.9–4.7)	8.9 (8.3–9.46)	14.1 (13.4–14.9)	16.7 (15.9–17.4)	24.7 (23.6–25.7)	5.7 (5.3–6.2)	6.8 (6.3–7.2)

Abbreviation: NA, not available.

a High blood pressure data were available only in 2013 and 2015.

b Significant difference compared with all workers in the survey.

**Table 3:** Adjusted Prevalence of Adverse Health Outcomes by Industry, Michigan Behavioral Risk Factor Surveillance System (MIBRFSS), 2013–2015

Standard Industrial Classification, Code	COPD Ever	Current Asthma	Asthma Ever	Depression Ever	High Blood Pressure Ever^a^	Diabetes Ever	Any Cancer Ever
	% (95% Confidence Interval)						
**Accommodation and Food Services, 72**	5.7 (3.4–7.9)	10.6 (7.5–13.7)	18 (13.9–22.0)	18.3 (14.4–22.2)	21.2 (16.3–26.1)	5.0 (2.9–7.0)	5.2 (2.5–7.9)
**Administrative, Support, and Waste Management, 56**	4.8 (0.8–8.7)	7.4 (2.6–12.3)	10.7 (5.5–15.9)	15.6 (10.4–20.8)	22.9 (16.1–29.6)	8 (3.2–12.7)	7.3 (3.7–11)
**Agriculture, Forestry, Fishing, and Hunting, 11**	NA	NA	8.9 (2.7–15.2)	6.7 (1.9–11.6)^b^	21.1 (12.8–29.5)	1.7 (0-3.5)	4.6 (1.8–7.4)
**Arts, Entertainment, and Recreation, 71**	NA	11.2 (5.9–16.4)	13.8 (8.1–19.5)	18.0 (10.5–25.5)	18.1 (11.7–24.5)	10.3 (6.0–14.6)	8.6 (4.6–12.7)
**Construction, 23**	2.6 (1.3–3.8)^b^	6.2 (3.3–9.1)^b^	10.6 (7.2–13.9)	13.0 (9.4–16.6)	24.0 (19.2–28.9)	3.7 (2.3–5.1)^b^	6.0 (3.8–8.2)
**Educational Services, 61**	3.9 (2.4–5.5)	11.4 (9.2–13.7)	16.3 (13.7–19.0)	16.2 (13.8–18.5)	28.1 (24.6–31.5)	5.8 (4.3–7.2)	7.0 (5.6–8.4)
**Finance and Insurance, 52**	5.1 (2.6–7.6)	9.3 (5.8–12.8)	15.6 (11.4–19.9)	19.6 (14.9–24.3)	27.2 (22.1–32.3)	7.5 (4.9–10.1)	7.7 (5.2–10.1)
**Health Care and Social Assistance, 62**	4.4 (3.3–5.5)	10.1 (8.4–11.7)	15.0 (13.0–17.0)	20.1 (17.9–22.4)^b^	27.2 (24.5–29.8)	6.5 (5.2–7.8)	7.4 (6.1–8.7)
**Information, 51**	4.0 (0.6–7.3)	10.7 (5.9–15.4)	16.7 (10.8–22.7)	21.9 (14.6–29.2)	24.6 (17.7–31.4)	4.1 (1.7–6.5)	7.2 (3.6–10.7)
**Manufacturing, 31**	4.3 (3.3–5.4)	6.7 (5.2–8.11)^b^	12.9 (11.0–14.9)	16.8 (14.7–19.0)	26.1 (23.7–28.6)	6.0 (4.9–7.1)	6.2 (5.1–7.4)
**Other Services (Except Public Administration), 81**	5.4 (3.4–7.4)	9.0 (5.6–12.4)	12.6 (8.9–16.3)	16.3 (12.8–19.8)	22.7 (18.6–26.9)	5.2 (3.3–7.2)	7.6 (5.4–9.7)
**Professional, Scientific, and Technical Service, 54**	4.7 (2.5–6.9)	11.2 (7.6–14.7)	15.9 (12.0–19.8)	14.2 (10.7–17.8)	27.2 (22.6–31.7)	6.7 (4.2–9.2)	6.7 (4.7–8.8)
**Public Administration, 56**	3.8 (2.2–5.5)	8.4 (5.5–11.3)	15.2 (11.1–19.2)	18.7 (14.5–22.8)	24.5 (20.1–28.9)	6.6 (4.3–8.8)	6.5 (4.5–8.4)
**Real Estate, Rental, and Leasing, 53**	2.2 (0.2–4.3)	8.2 (3.7–12.7)	12.4 (7.3–17.4)	16.6 (10.3–22.8)	28.7 (20.4–37.1)	9.6 (4.5–14.7)	5.5 (2.2–8.8)
**Retail Trade, 44**	4.7 (3.1–6.3)	7.6 (5.7–9.5)	13.9 (11.3–16.5)	17.5 (14.6–20.4)	26.6 (22.9–30.3)	4.4 (3.0–5.8)	7.1 (5.4–8.8)
**Transportation and Warehousing, 48**	5.0 (2.9–7.1)	7.7 (4.2–11.2)	13.8 (9.5–18.2)	11.5 (7.9–15.2)^b^	21 (16.5–25.6)	7.1 (4.5–9.7)	5.5 (3.3–7.8)
**Utilities, 22**	NA	7.9 (1.8–14)	9.4 (3.4–15.4)	17.1 (7.8–26.5)	29.6 (20.3–38.9)	4.1 (0.4–7.8)	6.0 (1.4–10.6)
**Wholesale Trade, 42**	NA	8.6 (4.0–13.2)	12.8 (6.9–18.7)	14.1 (8.6–19.6)	22.7 (13.4–32.0)	5.2 (2.4–7.9)	7.3 (4.5–10.2)
**All Industries**	4.4 (3.9–4.8)	8.8 (8.12–9.4)	14.1 (13.3–14.9)	17.0 (16.2–17.9)	25.4 (24.4–26.5)	5.8 (5.4–6.3)	6.7 (6.3–7.2)

Abbreviation: NA, not available.

a High blood pressure data were available only in 2013 and 2015.

b Significant difference compared with all workers in the survey.

Construction and Manufacturing had a significantly lower prevalence of current asthma compared with all workers but only in Manufacturing was the prevalence significantly lower after controlling for covariates. The significantly elevated prevalence in Accommodation and Food Services and in Health Care and Social Assistance did not persist after controlling for covariates (Tables 2 and 3). Accommodation and Food Services had a significantly increased prevalence of ever having asthma before but not after controlling for covariates (Tables 2 and 3).

Workers in Agriculture, Forestry, Fishing, and Hunting had the lowest prevalence of ever having depression (6.5%). Workers in 3 other industries also had significantly lower prevalence of depression compared with workers in all industries while workers in Accommodation and Food Services had a significantly higher prevalence (Table 2). Agriculture, Forestry, Fishing, and Hunting (6.7%) and Transportation and Warehousing (11.5%) continued to have a significantly decreased prevalence of depression whereas Health Care and Social Assistance had a significantly increased prevalence of depression (20.1%) after controlling for covariates, including sex (Table 3).

For cancer prevalence, the industry with the lowest prevalence of ever having any cancer was Accommodation and Food Services (3.0) (Table 2). Health Care and Social Assistance had a significantly higher prevalence. None of the cancer prevalence was significantly less than or elevated compared with prevalence in all industries, after controlling for covariates (Table 3).

Prevalence of ever having diabetes and of ever having high blood pressure prevalence were lower in 4 industries than in all industries combined and higher in one (Table 2). After controlling for covariates, the prevalence of diabetes remained decreased in Agriculture, Forestry, Fishing, and Hunting and was significantly decreased in Construction. (Table 3).

We found that 8 occupations had a significantly higher prevalence of ever having COPD, depression, high blood pressure, diabetes, or current asthma compared with workers in all occupations (Appendix 1), but only Protective Services had a significantly higher prevalence of high blood pressure (37.3%) and diabetes (12.8%) after adjusting for all confounding factors, and Construction and Extraction had a significantly lower prevalence of COPD (2.1%) (Appendix 2). We also calculated adjusted prevalences for ever having cardiovascular disease or arthritis by industry and occupation (Appendixes 3 and 4).

The prevalence of ever having high blood pressure was lower in the Accommodation and Food Services and in the Arts, Entertainment, and Recreation industries, and in the food preparation and serving-related occupations (Table 2). These lower prevalences were not significant after controlling for covariates (Table 3). The prevalence was only significantly elevated in the Protective Service occupation and persisted after controlling for covariates (Appendixes 1 and 2).

The prevalence of diabetes was significantly higher in the Arts, Entertainment, and Recreation industry and significantly lower in the Accommodation and Food Services industry and the Agriculture, Forestry, Fishing, and Hunting industry (Table 2). After controlling for covariates, the lower prevalence persisted in the Agriculture, Forestry, Fishing, and Hunting industry and became significantly lower in construction (Table 3). The diabetes prevalence was significantly higher in the Protective Service occupation (Appendix 1). Differences in prevalence in selected industries and occupations suggest the need for further work to identify workplace factors, such as shift work or noise, that may cause these differences.

## Discussion

Other states have examined differences in health outcomes by industry and occupation, but ours is the first analysis performed in Michigan. We waited to conduct analyses until we could combine 3 years of data to maximize the number of industry and occupation groups with sufficient sample size. However, even with 3 years’ worth of data, we did not have a sufficient sample size to separately present the results in 2 two-digit industry and 8 two-digit occupation groups. Besides being useful for Michigan prevention strategies, the analyses we conducted also add to previous literature that examined industry and occupation and BRFSS data by assessing prevalence by industry and occupation while controlling for important demographic factors, such as age, sex, and race, which are related to behaviors and health outcomes. Controlling for these demographic factors and behaviors, such as cigarette smoking, when examining health outcomes provides important information on interpreting whether differences in prevalence between industries and occupation are due to the health behaviors and demographics of the workforce in that industry or occupation or are possibly due to exposures or work practices associated with an industry or occupation.

Workers in Accommodation and Food Services, and in Health Care and Social Assistance had a significantly higher prevalence of current asthma, but not after controlling for covariates. Manufacturing had a significantly lower percentage that persisted after controlling for covariates. Construction and Extraction occupations and Production occupations had significantly lower prevalence of current asthma. The lower prevalence in Production occupations persisted whereas the prevalence in Health Care Support was no longer greater after controlling for covariates. Dodd and Mazurek (4) used 2013 BRFSS data from 21 states to examine the current asthma rate and found that the Health Care and Social Assistance industry had the highest prevalence, and education had the second highest prevalence (4). Michigan data also showed that education had the second highest prevalence of current asthma, although it was not significantly larger than the prevalence among all workers combined. By using 2006–2009 BRFSS data from the State of Washington, Anderson et al (3) found that 3 occupational groups — teachers (all levels), and counselors; administrative support workers, including clerical workers; and health service workers — had significantly higher prevalence ratios of current asthma than prevalence in all industries (3). Because of sample size limitations, we were unable to conduct analyses by the occupation subcategories used in Washington. The 3 occupations in the Washington analyses with elevated current asthma prevalence would have been included in our Management, Business, and Financial occupations, Sales and Related occupations, or Service occupations. None of our combined occupation categories had elevated prevalence of asthma after controlling for covariates. Neither of 2 previously published analyses of asthma analyses controlled for covariates (3,4). Results from the 21-state study and Michigan and Washington suggest industries and occupations that should be targeted for health education efforts regarding asthma management.

Analyses of the BRFSS data are useful to generate hypotheses for further research to explore differences between industries and occupations, to help focus primary prevention or secondary prevention action on certain worker groups, and to focus the priorities of industry worksite wellness programs. Better understanding of why certain industries and occupations have an increased prevalence of certain diseases despite controlling for known risk factors, such as cigarette smoking, may enable the identification of exposures or other modifiable risk factors. Simply comparing the prevalence without controlling for known risk factors can produce misleading interpretations. For example, depression and cancer are known to correlate highly with sex and age (9–11). After controlling for covariates, the only significant differences among industries and occupations for cancer or depression was a high prevalence of depression in the Health Care and Social Assistance industry while depression was significantly low in Agriculture, Forestry Fishing, and Hunting and in Transportation and Warehousing (Tables 2 and 3). Fan et al (6) examined the prevalence of current depression by occupation by using Washington 2006 and 2008 BRFSS data (6). They found that only truck drivers had significantly increased prevalence of depression after controlling for covariates. We found a lower prevalence of depression in Transportation and Warehousing, and in Utilities that persisted after controlling for covariates. The subcategory of truck driver could not be analyzed separately in the MIBRFSS database.

None of the differences between industries or occupations for ever having cancer persisted after adjusting for covariates and therefore were not useful in generating hypotheses to identify occupational carcinogens. The prevalence of cardiovascular disease was not significantly different in any industry or occupation either before or after adjustment despite differences in risk factors such as smoking, obesity, diabetes, and high blood pressure.

The analysis of health outcomes in conjunction with health behaviors is useful in considering alternative reasons for different findings by industry. For example, substantial differences in smoking prevalence were observed across different industries. COPD, which is highly associated with cigarette smoking, also differed among industries but not in the same way that cigarette smoking did (Construction had the highest prevalence of smoking and the lowest prevalence of COPD). Three possible explanations for why cigarette and COPD prevalence differ within the same industry are 1) workers in industries with a high cigarette but low COPD prevalence (eg, Construction) self-select out of the industry because of the strenuous work load, 2) industries with a low prevalence of smoking but a high prevalence of COPD (eg, Other Services) may have exposures that contribute to COPD, or 3) people with COPD move into this industry (eg, Other Services) once they develop COPD.

The inclusion of the industry and occupation questions in BRFSS provides information that allows both behaviors and health outcomes to be evaluated in relationship to work. We chose to present the results of the industry question in the main text because generally, it both provides a better measure of exposure and it identifies targets for interventions. We present data by occupation in the appendixes. Certain occupations, such as teachers, firefighters, and police, are specific enough to provide the same information as industry, but most occupations, such as cleaner, laborer, manager, and mechanic, are present in many different industries and do not facilitate public health interventions.

Our study had limitations. Despite widespread use to measure the prevalence of different diseases and behaviors at the state level, BRFSS data had limitations. First, health behaviors and outcomes were self-reported responses, and there was no review of medical records to check the validity of the responses. Second, results may not have been representative for the whole population, given a 50% response rate. Third, the data were cross-sectional, so the results were not evidence for causality but rather should be used to generate hypotheses.

We examined the adverse health outcomes and health behaviors among different industry sectors for MIBRFSS 2013–2015. Our results showed that 3 industries (Accommodation and Food Services; Arts, Entertainment, and Recreation; and Health Care and Social Assistance), which employ 24% of the Michigan workforce, had 7 significantly higher prevalence rates for adverse health outcomes: ever any cancer, depression, diabetes, and current asthma. Prevention efforts targeting these 3 industries have the greatest potential to reduce the overall burden of these common conditions in Michigan. Similar analyses in other states would allow prevention strategies to be uniquely targeted on a state-by-state basis.

## Appendix 1

**Table:** Distribution of Health Outcomes Among Employed Adults by Occupation: Michigan Behavioral Risk Factors Surveillance System (MIBRFSS), 2013–2015

Standard Occupational Classification, Code	COPD Ever	Current Asthma	Asthma Ever	Depression Ever	High Blood Pressure Ever^a^	Diabetes Ever	Any Cancer Ever
	Percentage (95% Confidence Interval)						
**Building and Grounds Cleaning and Maintenance, 37**	7.4 (4.8–10.0)	11.7 (8.2–15.2)	15.4 (11.3–19.4)	17.8 (13.7–21.9)	29.6 (23.5–35.7)	6.0 (3.3–8.7)	4.9 (2.7–7.0)
**Construction and Extraction, 47**	2.1 (1.0–3.1)^b^	4.7 (2.5–6.9)	10.6 (7.2–13.9^b^)	11.6 (8.5–14.7)^b^	25.9 (20.6–31.1)	4.3 (2.5–6.0)	4.1 (2.4–5.8)
**Food Preparation and Serving Related, 35**	7.5 (4.9–10.1)^b^	12.5 (9.1–15.9)	20.0 (15.7–24.2)	24.8 (20.0–29.5)^b^	14.7 (11.2–18.2)^b^	4.5 (2.8–6.3)	3.8 (2.4–5.3)
**Health Care Support, 29**	6.6 (3.4–9.8)	15.4 (10.8–20.0)^b^	22.1 (16.3–27.9)	26.3 (20.3–32.4)^b^	22.1 (16.1–28.0)	6.3 (3.4–9.2)	7.5 (4.4–10.7)
**Installation, Maintenance, and Repair Occupation, 49**	4.3 (2.4–6.2)	7.4 (4.0–10.8)	10.4 (6.5–14.2)^b^	10.6 (7.0–14.1)^b^	23.9 (18.7–29.2)	4.1 (2.3–5.8)	4.5 (2.6–6.4)^b^
**Management, Business, and Financial, 11, 13**	3.2 (2.2–4.2)	8.2 (6.7–9.8)	13.0 (11.1–14.9)^b^	14.2 (12.4–16.1)	26.1 (23.3–28.8)	7.0 (5.8–8.3)	8.6 (7.2–10)
**Office and Administrative Support, 43**	5.1 (3.8–6.3)	10.4 (8.6–12.3)	15.9 (13.7–18.2)	18.3 (15.9–20.7)	26.3 (23.2–29.3)	6.2 (4.9–7.4)	8.5 (7.0–10.0)
**Personal Care and Service, 39**	6.5 (3.7–9.4)	10.9 (7.6–14.3)	14.8 (11.0–18.6)	21.9 (17.1–26.7)	20.6 (15.5–25.7)	6.4 (4.0–8.8)	8.9 (6.0–11.8)
**Production, 51**	6.2 (4.4–7.9)	6.0 (4.1–7.8)^b^	12.6 (9.9–15.28)^b^	17.4 (14.5–20.3)	25.3 (21.4–29.2)	6.5 (4.8–8.2)	4.5 (3.0–6.0)
**Professional and Related, 15, 17, 19, 21, 23, 25, 27, 29**	2.4 (1.8–2.9)^b^	9.1 (8.1–10.2)	13.4 (12.2–14.7)^b^	15.8 (14.4–17.1)	23.5 (21.7–25.2)	5.1 (4.3–5.9)	7.5 (6.7–8.4)
**Protective Service, 33**	NA	NA	11.1 (6.2–15.9)	12.9 (8.2–17.6)	36.7 (28.5–44.9)^b^	12 (6.9–17.1)^b^	4.5 (1.9–7.1)
**Sales and Related, 41**	4.6 (3.4–5.8)	9.5 (7.5–11.5)	15.9 (13.5–18.4)	19.1 (16.4–21.8)	23.3 (20.1–26.5)	4.3 (3.2–5.4)	8.3 (6.8–9.7)
**Transportation and Material Moving, 53**	6.4 (4.1–8.7)	7.6 (5.1–10.1)	13.7 (10.4–16.9)	12.8 (9.9–15.8)	28.3 (23.4–33.2)	7.6 (5.1–10.1)	6.0 (4.0–8.0)
**All**	4.4 (3.9–4.8)	9.0 (8.4–9.6)	15.9 (15.3–16.5)	16.7 (15.9–17.5)	24.6 (23.5–25.6)	5.7 (5.3–6.2)	6.9 (6.4–7.3)

Abbreviation: NA, not available.

^a^ High blood pressure data were available only for 2013 and 2015.

^b^ Significant difference compared with workers employed in all occupations in MIBRFSS.

## Appendix 2

**Table:** Adjusted Prevalence of Adverse Health Outcomes by Occupation, Michigan Behavioral Risk Factor Surveillance System (MIBRFSS), 2013–2015

Standard Occupational Classification, Code	COPD Ever	Current Asthma	Asthma Ever	Depression Ever	High Blood Pressure Ever^a^	Diabetes Ever	Any Cancer Ever
	Percentage (95% Confidence Interval)						
**Building and Grounds Cleaning and Maintenance, 37**	5.7 (2.6–8.8)	10.6 (6–15.2)	13.6 (8.5–18.6)	15.3 (10.8–19.8)	28.4 (21.9–34.9)	6.3 (3.1–9.6)	5.8 (3.0–8.6)
**Construction and Extraction, 47**	2.1 (0.9–3.4)^b^	6.0 (2.8–9.1)	10.8 (7–14.5)^b^	13.5 (9.5–17.5)	25.7 (19.8–31.5)	4.1 (2.3–5.8)	5.3 (2.8–7.8)
**Food Preparation and Serving Related, 35**	5.5 (3.2–7.8)	11.6 (7.8–15.4)	17.7 (13.2–22.3)	18.3 (14.0–.022.6)	19.3 (14.5–24.2)^b^	6.5 (3.8–9.2)	5.5 (3.1–8.0)
**Health Care Support, 29**	5.6 (2.8–8.4)	11.9 (7.5–16.3)	18.6 (12.9–24.3)	21.4 (16.1–26.8)	27.4 (20.5–34.4)	9 (4.4–13.6)	10.4 (5.4–15.4)
**Installation, Maintenance, and Repair, 49**	4.3 (1.8–6.7)	9.5 (4.7–14.3)	11.5 (6.9–16.2)	12.8 (8.1–17.6)	23.3 (17.8–28.8)	3.7 (1.9–5.5)	5.9 (3.2–8.7)
**Management, Business, and Financial, 11, 13**	4.5 (2.9–6.1)	8.4 (6.5–10.2)	13.4 (11.1–15.6)	17.8 (15.3–20.2)	24.4 (21.6–27.1)	6.7 (5.4–8.1)	7.4 (6.0–8.9)
**Office and Administrative Support, 43**	4.2 (3.0–5.4)	9.8 (7.8–11.8)	15.9 (13.3–18.5)	16.1 (13.6–18.6)	27.8 (24.3–31.4)	6.1 (4.7–7.5)	7.3 (5.8–8.7)
**Personal Care and Service, 39**	4.8 (2.2–7.4)	8.9 (5.6–12.2)	12.2 (8.2–16.2)	16.6 (12.4–20.8)	23.6 (17.3–30.0)	6.8 (3.8–9.7)	8.9 (5.3–12.5)
**Production, 51**	4.1 (2.7–5.5)	5.9 (3.7–8.1)^b^	13.4 (10.0–16.7)	18.2 (14.8–21.7)	24.3 (20.2–28.4)	6.2 (4.4–8.1)	6.0 (3.9–8.1)
**Professional and Related, 15, 17, 19, 21, 23, 25, 27, 29**	3.7 (2.7–4.7)	9.2 (7.9–10.6)	14.0 (12.4–15.6)	18.1 (16.3–20.0)	25.0 (22.9–27.2)	5.6 (4.6–6.6)	6.5 (5.7–7.4)
**Protective service, 33**	NA	NA	13.3 (6.8–19.8)	19.1 (11.5–26.6)	37.3 (28.4–46.2)^b^	12.8 (7.2–18.3)^b^	5.1 (1.6–8.7)
**Sales and Related, 41**	4.5 (3.1–5.9)	8.0 (6.0–9.9)	15.1 (12.4–17.7)	17.4 (14.6–20.3)	24.3 (20.7–27.9)	4.2 (2.9–5.5)	7.3 (5.8–8.8)
**Transportation and Material Moving, 33**	6.8 (4.3–9.2)	8.7 (5.5–12)	15.0 (11.0–18.9)	13.8 (10.3–17.4)	25.6 (20.5–30.6)	6.1 (3.9–8.3)	7.0 (4.4–9.5)
**All **	4.4 (4.0–4.9)	8.9 (8.2–9.5)	14.2 (13.3–15.0)	17.1 (16.2–17.9)	25.3 (24.2–26.4)	5.9 (5.4–6.4)	6.8 (6.4–7.3)

Abbreviation: NA, not available.

^a^ High blood pressure data were only available in 2013 and 2015.

^b^ Significantly different from All occupations.

## Appendix 3

**Table:** Adjusted Prevalence of Asthma Ever, Current Asthma, and Arthritis by Industry, Michigan Behavioral Risk Factor Surveillance System (MIBRFSS), 2103–2015

Standard Industrial Classification, Code	Cardiovascular Disease Ever^a^	Arthritis Ever
	% (95% Confidence Interval)	
Accommodation and Food Services, 72	4.8 (2.4–7.2)	15.9 (12.7–19.2)
Administrative, Support, and Waste Management, 56	6.9 (2.8–10.9)	23.8 (18.4–29.3)
Agriculture, Forestry, Fishing, and Hunting, 11	2.6 (0.6–4.6)	20.9 (15.1–26.8)
Arts, Entertainment, and Recreation, 71	Not available	24.4 (17.8–31.0
Construction, 23,	3.5 (2.1–5.0)	20.3 (16.8–23.8)
Educational Services, 61	4.4 (3.1–5.7)	19.9 (17.7–22.2)
Finance and Insurance, 52	3.9 (2.0-5.8)	16.8 (13.4–20.2)
Health Care and Social Assistance, 62	3.9 (2.9–4.9)	20.9 (19.1–22.8)
Information, 51	Not available	14.4 (9.4–19.4)^b^
Manufacturing, 31	5.0 (3.9–6.1)	18.4 (16.6–20.2)
Other Services (Except Public Administration ), 81	4.1 (2.6–5.6)	19.7 (16.5–23.0)
Professional, Scientific, and Technical Service, 54	5.2 (2.8–7.6)	17.9 (14.8–21.1)
Public Administration, 56	4.9 (2.8–7.1)	21.7 (17.9–25.5)
Real Estate, Rental, and Leasing, 53	3.5 (1.5–5.4)	20.2 (15.0–25.5)
Retail Trade, 44	4.1 (2.8–5.4)	22.7 (20.1–25.4)
Transportation and Warehousing, 48	4.0 ((2.0–6.0)	20.1 (16.0–24.2)
Utilities, 22	Not available	18.0 (10.4–25.6)
Wholesale Trade, 42	3.5 (1.3–5.6)	17.4 (12.2–22.5)
All industries combined	4.3 (3.9–4.7)	20.1 (19.8–21.4)

^a^ A combination of ever having a heart attack, coronary heart disease, or a stroke.

^b^ Significant difference compared with workers employed in all occupations in MIBRFSS.

## Appendix 4

**Table:** Adjusted Prevalence of Cardiovascular Disease and Arthritis, by Occupation, Michigan Behavioral Risk Factor Surveillance System (MIBRFSS), 2013–2015

Standard Occupational Classification, Code	Cardiovascular Disease Ever^a^	Arthritis Ever
	% (95% Confidence Interval)	
Building and Grounds Cleaning and Maintenance, 37	6.4 (3.4–9.4)	23.7 (19.0–28.4)
Construction and Extraction Occupations, 47	4.2 (2.3–6.0)	20.7 (16.8–24.6)
Food Preparation and Serving Related, 35	4.6 (1.9–7.2)	15.5 (11.7–19.3)^b^
Health Care Support, 31	5.4 (1.6–9.3)	22.3 (17.4–27.1)
Installation, Maintenance, and Repair, 49	4.2 (2.0–6.5)	18.9 (14.8–23.0)
Management, Business, and Financial, 11, 13	3.5 (2.5–4.4)	22.6 (20.3–24.9)
Office and Administrative Support, 43	4.9 (3.4–6.4)	21.9 (19.6–24.2)
Personal Care and Service, 39	Not available	19.9 (15.7–24.2)
Production, 51	5.7 (3.9–7.5)	21.5 (18.5–24.4)
Professional and Related, 15, 17, 19, 21, 23, 25, 27, 29	3.9 (3.1–4.7	16.9 (15.6–18.1)
Protective Service, 33	5.6 (2.0–9.1)	19.4 (12.7–26.1)
Sales and Related, 41	4.1 (2.9–5.3)	19.9 (15.4–22.3)
Transportation and Material Moving, 53	3.7 (2.1–5.4)	19.8 (16.3–23.2)
All	4.3 (3.9–4.7)	20.7 (19.8–21.5)

^a^ A combination of every having a heart attack, coronary heart disease, or a stroke.

^b^ Significant difference compared with workers employed in all occupations in MIBRFSS.
